# Joinpoint trend analysis of prevalence of combustible and non-combustible tobacco product use by adults in the United States, using cross-sectional data from NHIS 2015–2023

**DOI:** 10.18332/tid/213343

**Published:** 2026-01-17

**Authors:** Yoonseo Mok, K. Michael Cummings, Colin W. Randol, Avery Roberson, David T. Levy, Rafael Meza

**Affiliations:** 1 Department of Epidemiology, University of Michigan, Ann Arbor, United States; 2 Department of Psychiatry and Behavioral Sciences, Medical University of South Carolina, Charleston, United States; 3 Department of Oncology, Lombardi Comprehensive Cancer Center, Georgetown University, Washington DC, United States; 4 Population Health Sciences, British Columbia Cancer Research Institute, Vancouver, Canada; 5 Rutgers Cancer Institute, Rutgers University, New Brunswick, United States

**Keywords:** tobacco prevalence, time trends, cigarettes, electronic cigarettes, smokeless tobacco

## Abstract

**INTRODUCTION:**

We describe population-level trends in the prevalence of any tobacco use, combustible and non-combustible tobacco use in US adults, by age and sex, from 2015 to 2023.

**METHODS:**

Cross-sectional data collected in the annual US National Health Interview Surveys (NHIS) conducted between 2015 and 2023 were used to characterize trends in adult current prevalence of any tobacco use, combustible and non-combustible tobacco product use. All NHIS participants with valid data on tobacco use, age and sex were included in the analyses. Joinpoint regression was used to estimate the annual percentage change (APC) from 2015 to 2023 and 95% confidence intervals (CIs) overall for each outcome and by different age groups (18–24, 25–34, 35–54, and ≥55 years) and for males and females. All statistical tests were two tailed and based on a p<0.05 significance level.

**RESULTS:**

Between 2015 and 2023 any tobacco use decreased at a statistically non-significant APC rate of -1.0% (95% CI: -2.1–0.12) across all age and sex groups combined. Overall, combustible tobacco decreased at a statistically significant APC rate of -3.6% (95% CI -4.6 – -2.6), with differences by age group (18–24: -12.2%, 95% CI: -14.6 – -9.7; 25–34: -5.2%, 95% CI: -6.6 – -3.8; 35–54: -2.5%, 95% CI: -4.2 – -0.9; ≥55: -1.2%, 95% CI: -1.8 – -0.5). Non-combustible tobacco product prevalence increased at a statistically significant APC of 8.9% (95% CI: 6.5–15.8) after 2017, with increases in all age groups and both sexes. The decrease in combustible tobacco was due to decreasing cigarette use while the increase in non-combustible tobacco products after 2017 was due to increases in electronic cigarette (EC) use.

**CONCLUSIONS:**

Tobacco products use by US adults shifted between 2015 and 2023, with combustible use decreasing, particularly in young adults, while non-combustible use increased.

## INTRODUCTION

Tobacco use patterns are changing in the United States (US), with decreases in cigarette smoking prevalence and increases in non-combustible forms of tobacco such as electronic cigarettes (ECs) and oral tobacco use^1^. Sales of non-combustible tobacco products are predicted to surpass the sales of combustible products in the next years, with revenues and profits from non-combustible tobacco products predicted to exceed those from smoked tobacco products in the next decade^1^.

While both combusted and non-combusted tobacco products pose health risks to users, they pose vastly different risks^2-5^. Factory-made cigarettes are the most dangerous tobacco product, responsible for about 480000 deaths annually in the US^5,6^. Given differential health risks of combusted and non-combusted tobacco products, the shifting patterns of use will likely have important impacts on future tobacco related premature deaths, with declines predicted over the next 50 years^7-9^. In this study, we describe population level trends in any tobacco use, combustible and non-combustible tobacco use in US adults, by age and sex, from 2015 to 2023.

## METHODS

### Study population and design

In this cross-sectional trend-analysis, we estimated the current use prevalence of combustible and non-combustible tobacco products using data from the National Health Interview Survey (NHIS) from 2015 to 2023^10^. The NHIS is a continuous, nationwide in-person survey of approximately 27000 adults annually that collects data on a wide range of health topics including information on tobacco product usage of the civilian, non-institutionalized US population. The NHIS uses multistage probability sampling that incorporates stratification, clustering, and oversampling of some subpopulations (e.g. Black, Hispanic, and Asian) in some years. Statistical weighting is used to generate representative national estimates^10^. The US Census Bureau employs and trains field staff to conduct interviews. They follow standardized procedures and conduct the majority of interviews in person, with some interviews done over the phone when an in-person household interview is not possible.

In this pooled secondary data analysis, we chose to examine trends in combustible and non-combustible tobacco product use starting in 2015, since the questions about tobacco use changed. In 2014, the NHIS survey combined the assessment of cigars and pipe use in a single question, whereas in 2015 separate questions were asked about cigars and pipe tobacco use. Previous studies have demonstrated that the formatting of questions about tobacco use can influence prevalence rates^11,12^. Harmonized NHIS data from 2015 to 2023 were obtained from IPUMS NHIS^10^.

### Independent variables

The primary independent variable in this study was survey year with nine time points spanning 2015 to 2023. In addition, we report tobacco use prevalence outcomes by age and sex. Age was categorized into four groups (18–24, 25–34, 35–54, and ≥55 years). Sex at birth was defined as either male or female. No other variables were considered in this descriptive study.

### Outcome variables

For each survey year, current tobacco use was characterized as: 1) any tobacco use, 2) combustible tobacco use; and 3) non-combustible tobacco use. Any tobacco product use was defined as use of either a combustible and/or non-combustible tobacco product. Combustible products used included cigarettes, cigars, pipe tobacco, and hookah tobacco. Non-combustible products used included electronic cigarettes and oral tobacco (i.e. chew, moist snuff, nicotine pouches). For each year, we defined people who currently reported smoking cigarettes as those who reported smoking 100 or more cigarettes in their lifetime and smoking every day (daily use) or some days (non-daily use). For other combustible tobacco products, current use was defined as any use of cigars, pipe and hookah tobacco in the past 30 days. Non-combustible tobacco product use included any current use of ECs and/or oral tobacco in the past 30 days.

### Analyses

Descriptive statistics including frequencies and percentages were generated using SAS 9.4 software (Cary, NC). We applied joinpoint regression to examine trends between 2015 and 2023 in: 1) weighted estimates of any tobacco use prevalence, 2) weighted estimates of combustible tobacco use prevalence, and 3) weighted estimates of non-combustible tobacco use prevalence. We report the annual percentage change (APC) from 2015 to 2023 and 95% confidence intervals (CIs) for males and females and combined, for all age groups and separately for the age groups: 18–24, 25–34, 35–54, and ≥55 years. Joinpoint regression also distinguishes different time segments (i.e. joinpoints) to identify significant differences between time points^13-15^. We allowed for at most one change in trend (0 or 1 joinpoints). All statistical tests were two-tailed and based on a p<0.05 significance level. The statistical tests are based on Kim et al.^14^ as implemented in the NCI joinpoint regression software^13^.

## RESULTS

**Figure 1:**
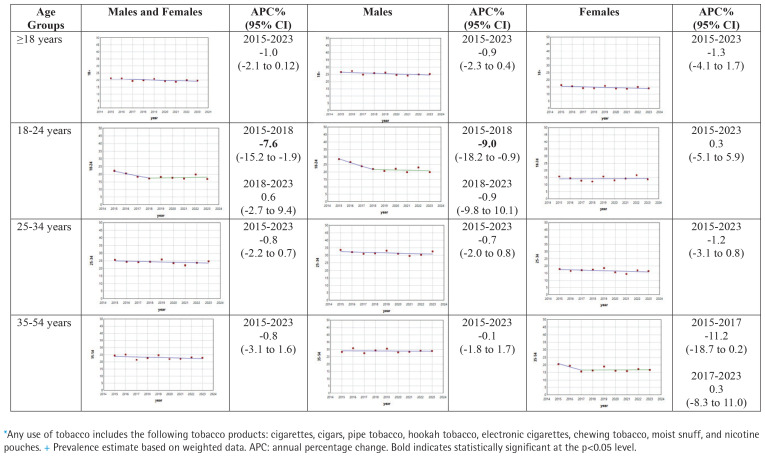
Joinpoint regression plots for the prevalence+ of any tobacco use*, by age and sex, for adult participants in the US NHIS 2015–2023

This pooled secondary data analysis includes 51791 respondents, 30565 males and 21226 females. Table 1 shows unweighted number of users, weighted prevalence (%) with 95% CIs for any tobacco use, among those ≥18 years and by different age groups, for males and females and combined, for the years 2015 to 2023. Figure 1 shows the results of the joinpoint analyses overall and by different age and sex. Between 2015 and 2023, across all ages and sex groups combined, any tobacco use decreased at a statistically non-significant APC rate of -1.0% (95% CI: -2.1–0.12). Among those aged 18–24 years, any tobacco use decreased at a statistically significant APC rate of -7.6% (95% CI: -15.2 – -1.9) between 2015 and 2018, but stabilized from 2018 to 2023 with an APC of 0.6% (95% CI: -2.7–9.4).

**Table 1:** Unweighted number (n) of people reporting any tobacco use* and weighted prevalence (%) estimates with 95% confidence intervals, by age and sex, for participants in the US NHIS 2015–2023

*Age (years)*	*2015*	*2016*	*2017*	*2018*	*2019*	*2020*	*2021*	*2022*	*2023*
**Males and Females**									
**≥18**									
Sample size, n	6903	7113	5410	5215	6218	5357	5250	4998	5327
Prevalence, %	21.2	21.1	19.3	19.8	20.8	19.1	18.8	19.8	19.5
95% CI	20.6–22.0	20.4–21.9	18.6–20.0	19.0–20.5	20.2–21.5	18.4–19.8	18.1–19.4	19.2–20.5	18.9–20.2
**18 – 24**									
Sample size, n	645	646	455	370	413	306	337	323	317
Prevalence, %	22.2	20.5	18.3	17.1	18.2	17.6	17.0	19.8	16.8
95% CI	20.1–24.4	18.4–22.7	16.3–20.3	14.9–19.4	16.2–20.1	15.4–19.8	15.1–19.0	17.5–22.1	14.8–18.9
**25 – 34**									
Sample size, n	1420	1301	1047	987	1156	922	950	888	985
Prevalence, %	25.6	24.3	24.0	24.3	25.8	23.4	22.0	23.6	24.6
95% CI	23.9–27.4	22.6–26.0	22.4–25.6	22.6–25.9	24.2–27.4	21.7–25.0	20.5–23.6	22.0–25.3	23.0–26.2
**35–54**									
Sample size, n	2646	2690	1936	1863	2332	1911	1906	1771	1855
Prevalence, %	24.4	25.0	21.4	22.7	24.6	21.9	22.0	23.1	22.8
95% CI	23.2–25.5	23.7–26.3	20.2–22.6	21.3–24.0	23.5–25.7	20.8–23.0	21.0–23.1	22.0–24.2	21.7–24.0
**≥55**									
Sample size, n	2192	2476	1972	1995	2317	2218	2057	2016	2170
Prevalence, %	15.7	16.2	15.5	15.9	16.0	15.2	15.1	15.4	15.4
95% CI	14.8–16.6	15.3–17.0	14.7–16.4	15.0–16.7	15.2–16.8	14.4–16.0	14.3–15.9	14.6–16.2	14.7–16.2
**Males**									
**≥18**									
Sample size, n	3933	4140	3187	3118	3693	3234	3110	2954	3196
Prevalence, %	26.5	27.2	24.8	25.9	26.2	24.6	24.2	25.0	25.2
95% CI	25.4–27.7	26.1–28.3	23.8–25.9	24.8–27.0	25.3–27.2	23.6–25.6	23.2–25.2	24.0–25.9	24.3–26.2
**18–24**									
Sample size, n	389	401	294	244	239	191	194	188	190
Prevalence, %	28.6	26.6	23.8	22.0	20.7	22.1	19.8	23.0	19.9
95% CI	25.3–31.8	23.4–29.8	20.7–26.9	18.6–25.3	17.7–23.6	18.8–25.5	16.8–22.9	19.7–26.3	16.9–22.8
**25–34**									
Sample size, n	840	759	637	593	713	603	595	555	618
Prevalence, %	33.6	32.1	31.0	31.3	33.1	31.0	29.6	30.3	32.6
95% CI	30.8–36.4	29.5–34.8	28.5–33.6	28.8–33.8	30.6–35.6	28.4–33.7	27.2–32.0	27.9–32.8	30.2–35.0
**35–54**									
Sample size, n	1478	1555	1154	1118	1380	1168	1154	1076	1106
Prevalence, %	28.4	30.8	27.5	29.3	30.6	28.1	28.4	29.2	29.0
95% CI	26.7–30.1	29.0–32.6	25.8–29.3	27.4–31.3	29.0–32.2	26.4–29.8	26.8–30.1	27.5–30.8	27.3–30.8
**≥55**									
Sample size, n	1226	1425	1102	1163	1361	1272	1167	1135	1282
Prevalence, %	20.2	21.2	19.5	21.2	20.7	19.1	19.2	19.4	20.2
95% CI	18.8–21.7	19.9–22.5	18.1–20.8	19.9–22.5	19.5–21.9	17.9–20.3	18.0–20.4	18.1–20.6	19.0–21.3
**Females**									
**≥18**									
Sample size, n	2970	2973	2223	2097	2525	2123	2140	2044	2131
Prevalence, %	16.3	15.5	14.2	14.1	15.8	14.0	13.7	15.0	14.1
95% CI	15.5–17.0	14.6–16.3	13.4–15.0	13.3–14.9	15.0–16.5	13.2–14.7	13.0–14.4	14.2–15.8	13.4–14.8
**18–24**									
Sample size, n	256	245	161	126	174	115	143	135	127
Prevalence, %	15.7	14.4	12.8	12.2	15.7	13.0	14.2	16.7	13.7
95% CI	13.0–18.4	12.0–16.8	10.1–15.5	9.5–14.8	13.1–18.3	10.3–15.8	11.7–16.8	13.5–19.8	11.1–16.3
**25–34**									
Sample size, n	580	542	410	394	443	319	355	333	367
Prevalence, %	17.9	16.6	17.1	17.4	18.5	15.6	14.4	17.0	16.5
95% CI	16.0–19.8	14.8–18.4	15.3–19.0	15.3–19.6	16.6–20.5	13.7–17.5	12.8–15.9	15.0–19.0	14.7–18.3
**35–54**									
Sample size, n	1168	1135	782	745	952	743	752	695	749
Prevalence, %	20.5	19.4	15.6	16.2	18.9	16.0	15.8	17.2	16.6
95% CI	19.0–22.0	17.8–21.0	14.2–16.9	14.8–17.7	17.5–20.3	14.7–17.4	14.6–17.1	15.8–18.5	15.3–17.9
**≥55**									
Sample size, n	966	1051	870	832	956	946	890	881	888
Prevalence, %	11.9	11.8	12.1	11.3	11.9	11.8	11.5	11.9	11.2
95% CI	10.9–12.8	10.9–12.7	11.2–13.1	10.4–12.3	11.0–12.8	10.9–12.8	10.7–12.4	11.0–12.8	10.3–12.0

* Any use of tobacco includes the following tobacco products: cigarettes, cigars, pipe tobacco, hookah tobacco, electronic cigarettes, chewing tobacco, moist snuff, and nicotine pouches.

**Figure 2:**
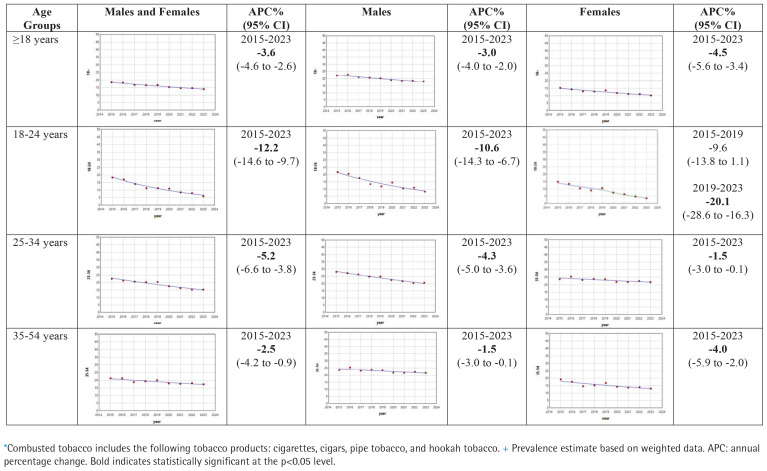
Joinpoint regression plots for the prevalence+ of combusted tobacco use*, by age and sex, for adult participants in the US NHIS 2015–2023

Table 2 shows unweighted number of users, weighted prevalence (%) with 95% CIs for combustible tobacco product use, among those ≥18 years and by different age groups, for males and females and combined, for the years 2015 to 2023. Figure 2 shows the results of the joinpoint analyses for combustible tobacco product use. Overall, combustible tobacco decreased at a statistically significant APC rate of -3.6% (95% CI: -4.6 – -2.6), with differences by age group (18–24 years: -12.2%, 95% CI: -14.6 – -9.7; 25–34 years: -5.2%, 95% CI: -6.6 – -3.8; 35–54 years: -2.5%, 95% CI: -4.2 – -0.9; and ≥55 years: -1.2%, 95% CI: -1.8 – -0.5). For females, the magnitude of the decline in combustible tobacco use prevalence was most apparent in younger age groups (i.e. aged 18–34 years) after 2019.

**Table 2:** Unweighted number (n) of people reporting combusted tobacco use* and weighted prevalence (%) estimates with 95% confidence intervals, by age and sex, for participants in the US NHIS 2015–2023

*Age (years)*	*2015*	*2016*	*2017*	*2018*	*2019*	*2020*	*2021*	*2022*	*2023*
**Males and Females**									
**≥18**									
Sample size, n	6150	6190	4719	4455	5087	4383	4203	3871	3984
Prevalence, %	18.5	18.4	16.7	16.6	16.8	15.2	14.6	14.6	14.0
95% CI	17.9–19.1	17.7–19.1	16.1–17.4	15.9–17.2	16.2–17.3	14.6–15.8	14.0–15.1	14.1–15.2	13.5–14.5
**18–24**									
Sample size, n	547	536	354	250	260	186	170	129	119
Prevalence, %	18.3	16.9	14.0	11.2	11.2	10.9	8.5	7.9	5.9
95% CI	16.3–20.2	14.9–19.0	12.2–15.8	9.3–13.1	9.7–12.8	9.1–12.7	7.1–9.8	6.3–9.6	4.7–7.2
**25–34**									
Sample size, n	1250	1134	901	823	900	680	697	584	599
Prevalence, %	22.5	21.3	20.6	20.2	20.3	17.4	16.2	15.2	15.2
95% CI	20.9–24.1	19.7–22.9	19.0–22.1	18.7–21.7	18.9–21.7	15.9–19.0	14.8–17.5	13.8–16.5	14.0–16.5
**35–54**									
Sample size, n	2349	2303	1680	1603	1893	1566	1534	1396	1401
Prevalence, %	21.3	21.3	18.7	19.3	20.0	17.9	17.6	18.1	17.3
95% CI	20.2–22.4	20.1–22.5	17.6–19.8	18.1–20.5	19.0–21.1	16.8–18.9	16.6–18.6	17.0–19.1	16.2–18.3
**≥55**									
Sample size, n	2004	2217	1784	1779	2034	1951	1802	1762	1865
Prevalence, %	14.1	14.7	14.0	14.1	14.0	13.2	13.1	13.5	13.2
95% CI	13.3–14.9	13.8–15.5	13.2–14.8	13.3–14.9	13.2–14.7	12.5–14.0	12.4–13.8	12.7–14.2	12.5–13.9
**Males**									
≥**18**									
Sample size, n	3346	3414	2670	2534	2871	2536	2400	2263	2344
Prevalence, %	22.1	22.7	20.9	20.6	20.1	18.9	18.2	18.4	18.0
95% CI	21.1–23.1	21.7–23.8	20.0–21.8	19.7–21.6	19.3–21.0	18.0–19.8	17.4–19.1	17.6–19.2	17.2–18.8
**18 – 24**									
Sample size, n	308	309	220	156	141	121	103	89	82
Prevalence, %	21.6	20.5	17.7	13.4	11.9	14.4	10.5	10.9	8.2
95% CI	18.8–24.4	17.4–23.5	14.9–20.4	10.7–16.1	9.6–14.1	11.6–17.3	8.3–12.7	8.4–13.4	6.2–10.3
**25 – 34**									
Sample size, n	698	620	535	467	552	429	426	376	380
Prevalence, %	28.0	27.2	26.4	24.7	24.8	22.4	21.6	20.2	20.6
95% CI	25.4–30.7	24.7–29.7	23.9–28.8	22.4–27.0	22.5–27.1	20.0–24.8	19.4–23.8	18.1–22.4	18.6–22.6
**35–54**									
Sample size, n	1236	1258	951	911	1051	909	893	830	820
Prevalence, %	23.5	25.3	23.1	23.7	23.5	21.7	21.7	22.2	21.6
95% CI	21.9–25.1	23.6–26.9	21.5–24.7	21.9–25.5	21.9–25.0	20.1–23.2	20.3–23.1	20.7–23.8	20.1–23.1
≥**55**									
Sample size, n	1104	1227	964	1000	1157	1077	978	968	1062
Prevalence, %	17.7	18.7	17.1	18.2	17.5	16.1	16.0	16.5	16.7
95% CI	16.4–19.0	17.5–20.0	15.9–18.3	16.9–19.4	16.4–18.6	14.9–17.2	14.8–17.1	15.3–17.7	15.7–17.8
**Females**									
≥**18**									
Sample size, n	2804	2776	2049	1921	2216	1847	1803	1608	1640
Prevalence, %	15.2	14.3	12.9	12.8	13.6	11.8	11.1	11.0	10.2
95% CI	14.5–15.9	13.5–15.1	12.1–13.7	12.0–13.5	12.9–14.3	11.1–12.5	10.5–11.7	10.3–11.7	9.6–10.8
**18 – 24**									
Sample size, n	239	227	134	94	119	65	67	40	37
Prevalence, %	14.8	13.4	10.4	9.0	10.6	7.4	6.4	4.9	3.6
95% CI	12.2–17.4	11.0–15.8	8.1–12.6	6.6–11.4	8.4–12.8	5.4–9.5	4.7–8.1	3.1–6.7	2.4–4.9
**25 – 34**									
Sample size, n	552	514	366	356	378	251	271	208	219
Prevalence, %	17.1	15.5	14.8	15.8	15.8	12.4	10.7	10.1	9.8
95% CI	15.3–19.0	13.7–17.3	13.0–16.5	13.8–17.8	14.0–17.5	10.7–14.2	9.3–12.2	8.6–11.7	8.4–11.2
**35–54**									
Sample size, n	1113	1045	729	692	842	657	641	566	581
Prevalence, %	19.1	17.5	14.5	15.0	16.7	14.2	13.6	13.9	13.0
95% CI	17.7–20.6	16.1–19.0	13.2–15.9	13.7–16.4	15.4–18.0	12.9–15.5	12.5–14.8	12.6–15.1	11.8–14.1
≥**55**									
Sample size, n	900	990	820	779	877	874	824	794	803
Prevalence, %	11.0	11.2	11.4	10.6	11.0	10.8	10.6	10.8	10.1
95% CI	10.0–11.9	10.3–12.1	10.5–12.4	9.7–11.5	10.1–11.8	9.9–11.7	9.8–11.4	9.9–11.7	9.3–10.9

* Combusted tobacco includes the following tobacco products: cigarettes, cigars, pipe tobacco, and hookah tobacco.

**Figure 3:**
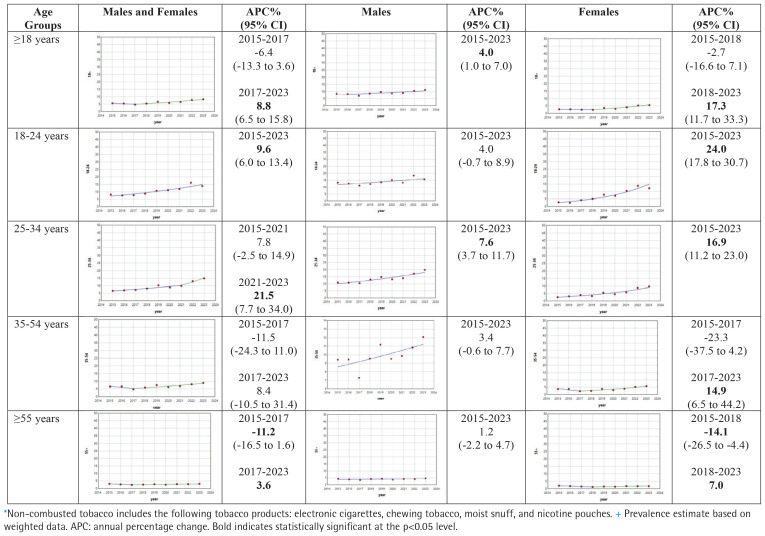
Joinpoint regression plots for the prevalence+ of non-combusted tobacco use*, by age and sex, for adult participants in the US NHIS 2015–2023

Table 3 shows unweighted number of users, weighted prevalence (%) with 95% CIs for non-combustible tobacco product use, among those ≥18 years and by different age groups, for males and females and combined, for the years 2015 to 2023. Figure 3 shows the results of the joinpoint analyses for non-combustible tobacco product use. Overall, non-combustible tobacco product prevalence increased at a statistically significant APC of 8.9% (95% CI: 6.5–15.8) after 2017, with increases in all age groups and both sexes, but with larger increases in younger age groups (i.e. 18–34 years).

**Table 3:** Unweighted number of people reporting non-combusted tobacco use*, and weighted prevalence (%) estimates with 95% confidence intervals, by age and sex, for participants in the US NHIS 2015–2023

*Year of survey*	*2015*	*2016*	*2017*	*2018*	*2019*	*2020*	*2021*	*2022*	*2023*
**Males and females**									
**≥18 years**									
Sample size, n	1658	1785	1287	1363	1843	1436	1632	1734	2017
Prevalence, %	5.6%	5.4%	4.7%	5.4%	6.6%	5.8%	6.5%	7.8%	8.3 %
95% CI	5.1–6.0	5.1–5.8	4.4–5.1	5.1–5.8	6.2–7.0	5.5–6.2	6.1–6.9	7.4–8.3	7.9–8.8
**18 –24 years**									
Sample size, n	220	232	191	191	233	191	232	262	260
Prevalence, %	8.1%	7.6%	7.7%	8.7%	10.7%	11.1%	11.8%	16.0%	13.6 %
95% CI	6.7–9.5	6.2–8.9	6.2–9.2	7.2–10.3	9.1–12.3	9.3–12.9	10.1–13.6	14.0–18.1	12.0–15.7
**25–34 years**									
Sample size, n	370	368	315	334	470	370	425	472	590
Prevalence, %	6.6%	6.9%	7.2%	8.1%	10.2%	8.9%	9.9%	13.0%	14.9 %
95% CI	5.7–7.6	6.0–7.9	6.2–8.2	7.2–9.1	9.1–11.3	7.8–10.0	8.8–11.0	11.7–14.2	13.5–16.1
**35–54 years**									
Sample size, n	670	716	460	496	720	532	590	616	718
Prevalence, %	6.5%	6.6%	4.8%	5.9%	7.5%	6.2%	6.9%	8.0%	8.9 %
95% CI	5.8–7.2	6.0–7.3	4.2–5.3	5.3–6.6	6.9–8.1	5.6–6.8	6.2–7.5	7.3–8.8	8.2–9.7
≥**55 years**									
Sample size, n	398	469	321	342	420	343	385	384	449
Prevalence, %	3.2%	2.8%	2.5%	2.7%	2.9%	2.5%	3.0%	3.0%	3.2 %
95% CI	2.7–3.7	2.5–3.2	2.1–2.8	2.3–3.0	2.6–3.2	2.2–2.9	2.6–3.3	2.6–3.3	2.8–3.5
**Males**									
≥**18 years**									
Sample size, n	1145	1281	913	1019	1293	1027	1067	1103	1288
Prevalence, %	8.5%	8.2%	7.0%	8.6%	9.7%	8.8%	8.9%	10.4%	11.2 %
95% CI	7.8–9.2	7.6–8.8	6.4–7.6	8.0–9.3	9.0–10.3	8.1–9.4	8.3–9.6	9.7–11.1	10.4–11.9
**18–24 years**									
Sample size, n	169	186	140	138	148	126	128	150	150
Prevalence, %	13.2%	12.6%	11.1%	12.2%	13.5%	15.1%	13.2%	18.3%	15.6 %
95% CI	10.6–15.8	10.1–15.1	8.8–13.5	9.7–14.6	11.0–16.0	12.2–18.1	10.6–15.9	15.3–21.3	13.1–18.1
**25–34 years**									
Sample size, n	287	281	230	257	343	272	283	304	382
Prevalence, %	10.8%	10.8%	10.4%	13.0%	14.7%	13.2%	13.9%	17.2%	19.8 %
95% CI	9.1–12.6	9.1–12.4	8.8–12.0	11.3–4.8	12.9–16.5	11.4–15.0	12.2–15.6	15.2–19.2	17.7–21.9
**35–54 years**									
Sample size, n	457	509	335	381	519	391	395	401	453
Prevalence, %	9.4%	9.4%	7.3%	9.5%	11.2%	9.5%	9.9%	10.8%	12.1 %
95% CI	8.2–10.6	8.3–10.5	6.3–8.3	8.4–10.7	10.1–12.2	8.5–10.6	8.8–10.9	9.7–12.0	10.8–13.3
≥**55 years**									
Sample size, n	232	305	208	243	283	238	261	248	303
Prevalence, %	4.5%	4.0%	3.5%	4.3%	4.4%	3.8%	4.3%	4.3%	4.7 %
95% CI	3.6–5.3	3.5–4.6	2.9–4.2	3.6–4.9	3.8–5.1	3.2–4.4	3.7–4.9	3.7–4.9	4.1–5.4
**Females**									
≥**18 years**									
Sample size, n	513	504	374	344	550	409	565	631	729
Prevalence, %	2.8%	2.8%	2.6%	2.5%	3.7%	3.1%	4.2%	5.4%	5.6 %
95% CI	2.5–3.2	2.5–3.2	2.2–2.9	2.2–2.8	3.3–4.1	2.7–3.5	3.8–4.6	4.9–5.9	5.1–6.1
**18–24 years**									
Sample size, n	51	46	51	53	85	65	104	112	110
Prevalence, %	2.3%	2.6%	4.3%	5.1%	7.8%	7.1%	10.4%	13.7%	12.1 %
95% CI	1.9–3.8	1.7–3.4	2.5–6.1	3.4–6.8	6.0–9.7	5.0–9.2	8.2–12.7	10.9–16.6	9.6–14.6
**25–34 years**									
Sample size, n	83	87	85	77	127	98	142	168	208
Prevalence, %	2.6%	3.2%	4.1%	3.3%	5.6%	4.6%	5.8%	8.7%	9.7 %
95% CI	1.8–3.4	2.2–4.1	3.0–5.1	2.5–4.2	4.5–6.7	3.5–5.7	4.8–6.9	7.3–10.2	8.3–11.2
**35–54 years**									
Sample size, n	213	207	125	115	201	141	195	215	265
Prevalence, %	3.7%	3.9%	2.3%	2.5%	3.9%	3.1%	4.0%	5.2%	5.8 %
95% CI	3.1–4.4	3.1–4.7	1.9–2.8	2.0–3.0	3.3–4.6	2.5–3.7	3.3–4.7	4.4–6.0	5.0–6.6
**≥55 years**									
Sample size, n	166	164	113	99	137	105	124	136	146
Prevalence, %	2.1%	1.8%	1.6%	1.3%	1.6%	1.4%	1.9%	1.8%	1.8 %
95% CI	1.7–2.6	1.5–2.2	1.2–1.9	1.0–1.6	1.3–1.9	1.0–1.8	1.5–2.2	1.4–2.1	1.5–2.2

* non–combusted tobacco includes the following tobacco products electronic cigarettes, chewing tobacco, moist snuff, nicotine pouches.

Supplementary file Tables 1 and 2 show unweighted number of users, weighted prevalence (%) with 95% CIs for cigarettes and non-cigarette combustible tobacco product use by survey year, age, and sex. Supplementary file Figures 1 and 2 show the results of the joinpoint analyses for cigarettes and non-cigarette combustible tobacco use. The results show that decreasing combustible tobacco products use prevalence is due to declining cigarette use.

Supplementary file Tables 3 and 4 show unweighted number of users, weighted prevalence (%) with 95% CIs for ECs and oral tobacco product use by survey year, age, and sex. For oral tobacco product use, the results are restricted to males and females combined, and for males, since the number of exclusive female users of oral tobacco was too low to reliably estimate prevalence rates. Supplementary file Figures 3 and 4 show the results of the joinpoint analyses for ECs and oral tobacco products trends. The results reveal increasing prevalence of EC use among both males and females after 2017. At least up until 2023, the use of oral tobacco products declined in nearly all age groups except for those aged ≥55 years.

## DISCUSSION

Overall adult use of any tobacco products remained stagnant between 2015 and 2023. However, the overall statistically non-significant trend in any tobacco use masks important changes in the types of tobacco products consumers are using. Combustible tobacco product use decreased annually among males and females, but the decline was much more apparent among younger adults, which coincides with decreasing cigarette smoking prevalence. Conversely, non-combustible product use increased significantly after 2017, especially among younger adults, initially among males and followed by females, due to increased use of ECs. While the decline in combustible tobacco product use is good news, the rate of decline was much less among those aged ≥35 years, where cigarette use remained high and only slowly declined between 2015 and 2023.

The rapid increase in non-combustible tobacco use among younger adults reflects the growing popularity of ECs and supports the idea that ECs are replacing cigarettes as the main type of tobacco product consumed^1,9^. In 2023, the prevalence of EC use was greater than cigarettes among adults aged 18–24 years. While non-combustible tobacco product use can cause nicotine dependence and is not completely safe, the absence of burned tobacco reduces exposure to harmful and potentially harmful chemicals compared to combustible tobacco use, especially cigarettes^16-19^.

### Limitations

While the findings from this study provide a broad non-causal descriptive assessment of population trends in tobacco product use, these findings should not be over interpreted as being predictive of future tobacco-related disease rates, for several reasons. First, we are only reporting the prevalence of current use defined as use in the past 30 days which fails to capture frequency, intensity and duration of product use all of which are important determinants of future health risks^5^. Second, self-reported tobacco use is prone to information bias and misclassification. For example, the negative stigma associated with smoking cigarettes has been found to be associated with underreporting smoking in surveys^20^. Also, not all respondents will be aware of the different tobacco products asked about in the survey, which may contribute to measurement error. Third, we only report cross-sectional population level prevalence rates and do not consider individual patterns of tobacco product use which are likely complex with people transitioning overtime between different tobacco products use states, including using more than one product at a time (i.e. dual use)^21-27^. For example, studies of people who report currently using cigarettes have found that most are still smoking cigarettes for a year or even five years, suggesting that different tobacco products might convey different risks of abuse liability^23-28^. Fourth, there are inherent limitations of the joinpoint analyses presented in this study, since we use only nine data points. It is possible results will change as additional data points become available. Fifth, the US findings from this study may not apply to other countries.

## CONCLUSIONS

This study found that the types of tobacco products used by US adults has shifted between 2015 and 2023. Combustible use – especially cigarettes – is decreasing, particularly in young adults, while non-combustible use is increasing. Continued monitoring of tobacco product use trends is warranted to understand market dynamics, identify priorities for regulatory and tobacco control policies, and to attempt to predict future tobacco-related deaths.

## Supplementary Material



## Data Availability

The data supporting this research are available from the following link: https://healthsurveys.ipums.org/
