# Supplementary material for: Joinpoint trend analysis of prevalence of combustible and non-combustible tobacco product use by adults in the United States, using cross-sectional data from NHIS 2015–2023
Source: Tob Induc Dis. 2026 Jan 17;24:10.18332/tid/213343. doi: 10.18332/tid/213343 (PMC12811843; doi:10.18332/tid/213343)
Supplement: Supplementary file 1 [file TID-24-05-s1.pdf]

Supplementary Figure 1. Joinpoint regression plots for the prevalence[+] of cigarette use, by age and sex, for adult participants in the US NHIS 2015-2025

| Age Groups | Males and Females | APC% (95% CI) | Males | APC% (95% CI) | Females | APC% (95% CI) |
|---|---|---|---|---|---|---|
| ≥18 years | 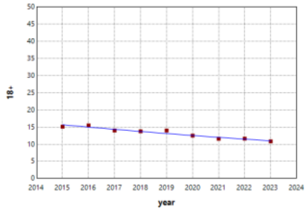 | 2015-2023<br>**-4.3**<br>(-5.6 to -3.2) | 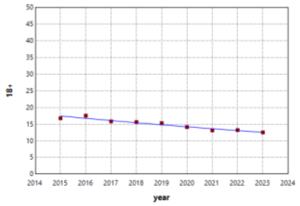 | 2015-2023<br>**-4.1**<br>(-5.0 to -3.1) | 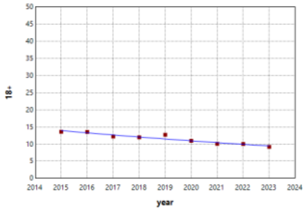 | 2015-2023<br>**-4.7**<br>(-6.2 to -3.3) |
| 18-24 years | 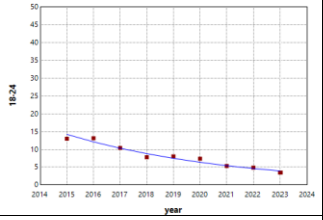 | 2015-2023<br>**-14.8**<br>(-18.3 to -11.1) | 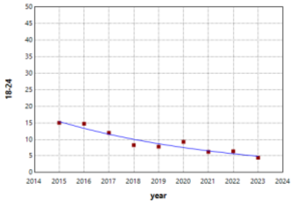 | 2015-2023<br>**-13.3**<br>(-17.8 to -8.6) | 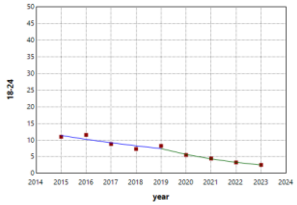 | 2015-2019<br>-10.1<br>(-14.7 to 0.5)<br><br>2019-2023<br>**-23.6**<br>(-31.5 to -19.3) |
| 25-34 years | 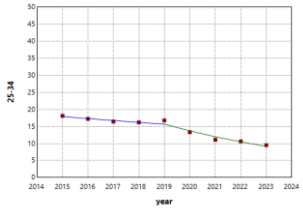 | 2015-2019<br>-3.3<br>(-6.0 to 2.0)<br><br>2019-2023<br>**-12.6**<br>(-17.2 to -9.9) | 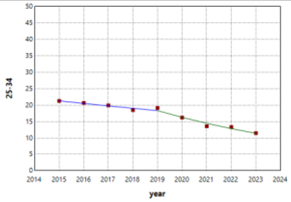 | 2015-2019<br>**-3.7**<br>(-5.9 to -0.1)<br><br>2019-2023<br>**-11.2**<br>(-14.7 to -9.1) | 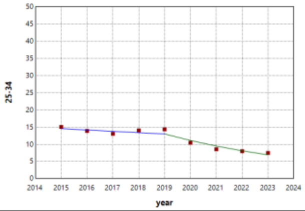 | 2015-2019<br>-2.8<br>(-7.8 to 15.7)<br><br>2019-2023<br>**-14.6**<br>(-29.1 to -9.9) |
| 35-54 years | 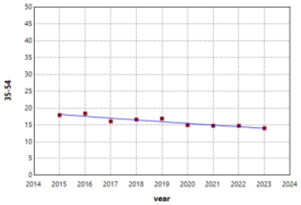 | 2015-2023<br>**-3.2**<br>(-4.4 to -1.9) | 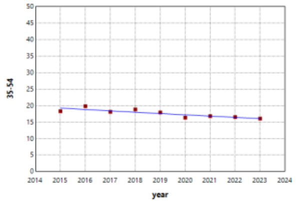 | 2015-2023<br>**-2.2**<br>(-3.5 to -0.9) | 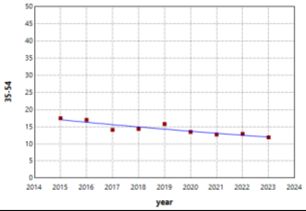 | 2015-2023<br>**-4.3**<br>(-6.4 to -2.1) |
| ≥55 years | 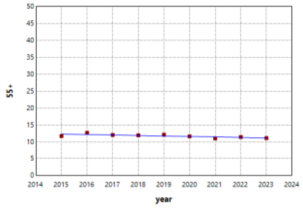 | 2015-2023<br>**-1.2**<br>(-2.0 to -0.4) | 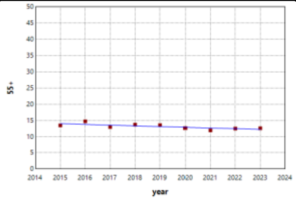 | 2015-2023<br>-1.7<br>(-3.5 to 0.3) | 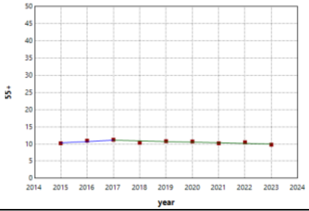 | 2015-2017<br>3.6<br>(-2.0 to 10.1)<br><br>2017-2023<br>-1.7<br>(-7.9 to 1.3) |

[+]Prevalence estimates based on weighted data
Note: **Bold** indicates significant at the p<0.05 level.

Supplementary Figure 2. Joinpoint regression plots for the prevalence[+] of other combustible tobacco product use[*], by age and sex, for adult participants in the US NHIS 2015-2025

| **Age Groups** | **Males and Females** | **APC% (95% CI)** | **Males** | **APC% (95% CI)** | **Females** | **APC% (95% CI)** |
|---|---|---|---|---|---|---|
| ≥18 years | 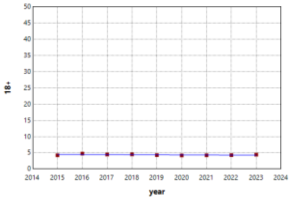 | 2015-2023<br>-0.6<br>(-2.2 to 1.0) | 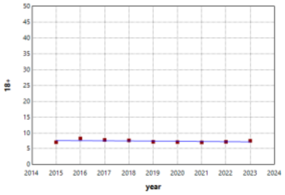 | 2015-2023<br>-0.7<br>(-2.8 to 1.4) | 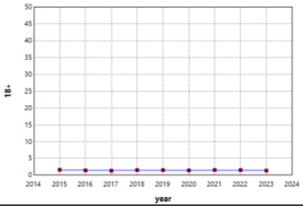 | 2015-2023<br>-0.5<br>(-2.5 to 1.6) |
| 18-24 years | 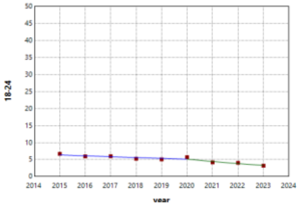 | 2015-2020<br>-4.3<br>(-10.5 to 12.9)<br><br>2020-2023<br>**-13.9**<br>(-28.1 to -5.3) | 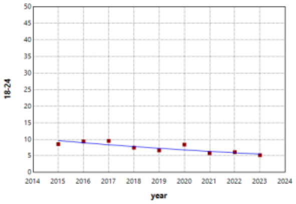 | 2015-2023<br>**-6.6**<br>(-11.1 to -1.9) | 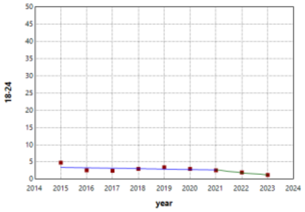 | 2015-2021<br>-4.0<br>(-12.4 to 22.5)<br><br>2021-2023<br>**-32.2**<br>(-48.5 to -9.4) |
| 25-34 years | 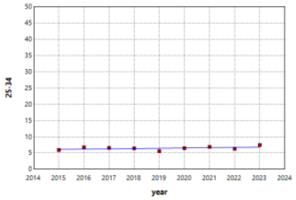 | 2015-2023<br>1.3<br>(-1.2 to 4.0) | 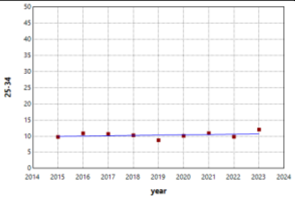 | 2015-2019<br>0.9<br>(-1.9 to 3.9) | 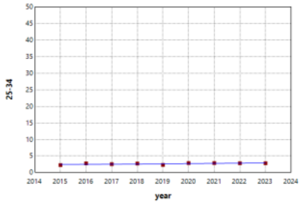 | 2015-2023<br>2.2<br>(-0.5 to 5.0) |
| 35-54 years | 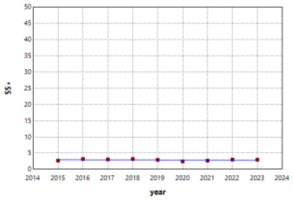 | 2015-2023<br>1.2<br>(-1.3 to 3.7) | 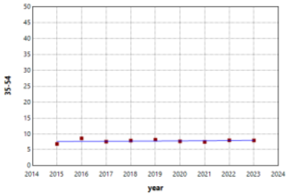 | 2015-2023<br>0.5<br>(-1.2 to 2.3) | 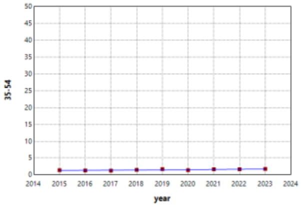 | 2015-2023<br>**3.8**<br>(0.3 to -7.5) |
| ≥55 years | 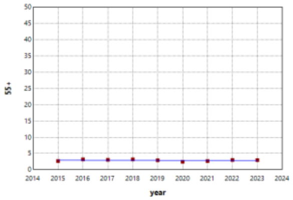 | 2015-2023<br>-0.5<br>(-3.0 to 2.1) | 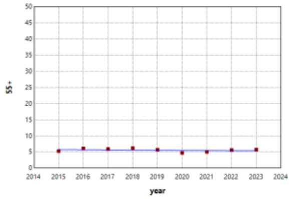 | 2015-2023<br>-0.8<br>(-3.2 to 1.7) | 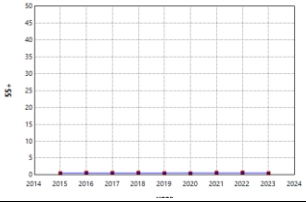 | 2015-2023<br>0.4<br>(-6.5 to 7.9) |

[+]Prevalence estimates based on weighted data

[*]Other combustible tobacco use includes cigars, pipe tobacco, and hookah tobacco
Note: **Bold** indicates significant at the p<0.05 level.

Supplementary Figure 3. Joinpoint regression plots for the prevalence[+] of electronic cigarette use, by age and sex, for adult participants in the US NHIS 2015-2025

| Age Groups | Males and Females | APC% (95% CI) | Males | APC% (95% CI) | Females | APC% (95% CI) |
|---|---|---|---|---|---|---|
| ≥18 years | 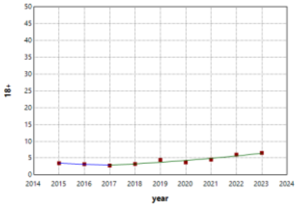 | 2015-2017<br>-9.2<br>(-18.8 to 5.1)<br><br>2017-2023<br>**14.5**<br>(11.1 to 23.3) | 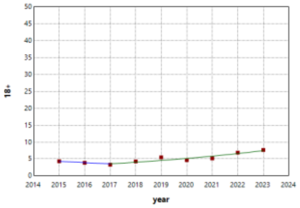 | 2015-2017<br>-9.0<br>(-19.9 to 9.4)<br><br>2017-2023<br>**13.2**<br>(7.1 to 31.8) | 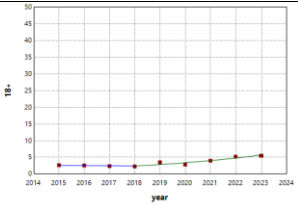 | 2015-2018<br>-3.0<br>(-17.5 to 7.4)<br><br>2018-2023<br>**18.9**<br>(13.2 to 35.1) |
| 18-24 years | 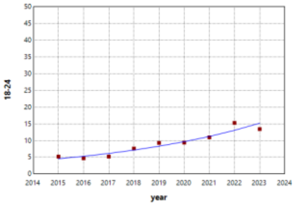 | 2015-2023<br>**16.3**<br>(9.2 to 24.0) | 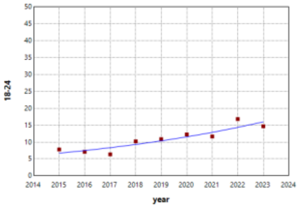 | 2015-2023<br>**11.5**<br>(5.3 to 17.9) | 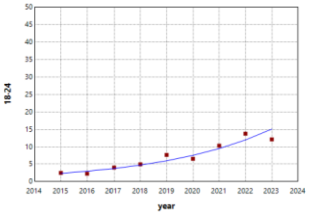 | 2015-2023<br>**26.0**<br>(19.8 to 32.6) |
| 25-34 years | 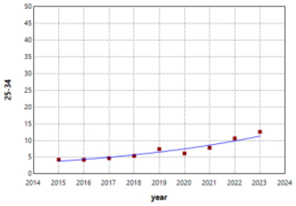 | 2015-2023<br>**14.7**<br>(7.6 to 22.3) | 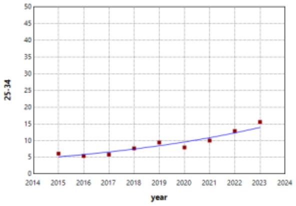 | 2015-2023<br>**13.3**<br>(6.9 to 20.1) | 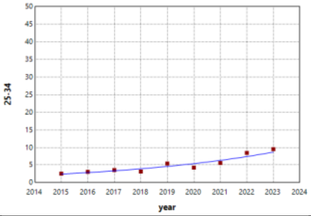 | 2015-2023<br>**17.2**<br>(12.7 to 21.9) |
| 35-54 years | 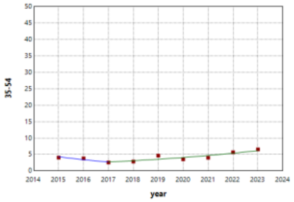 | 2015-2017<br>-20.9<br>(-38.3 to 14.3)<br><br>2017-2023<br>14.9<br>(-14.0 to 57.3) | 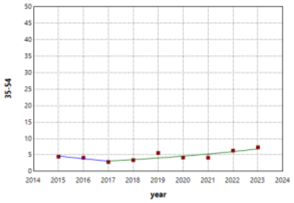 | 2015-2017<br>-18.0<br>(-34.8 to 11.5<br><br>2017-2023<br>13.8<br>(-5.5 to 47.8) | 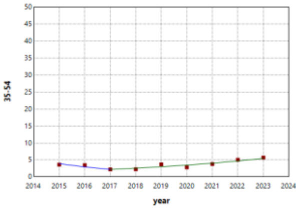 | 2015-2017<br>-24.5<br>(-38.3 to 3.7)<br><br>2017-2023<br>**16.2**<br>(7.8 to 45.6) |
| ≥55 years | 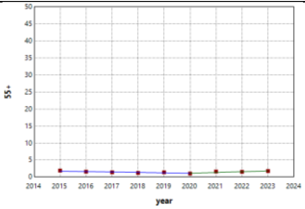 | 2015-2020<br>-8.1<br>(-28.5 to 1.6)<br><br>2020-2023<br>16.8<br>(-2.7 to 50.6) | 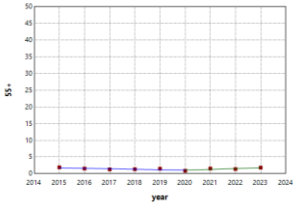 | 2015-2020<br>**-9.6**<br>(-28.2 to -1.3)<br><br>2020-2023<br>**18.9**<br>(0.3 to 53.8) | 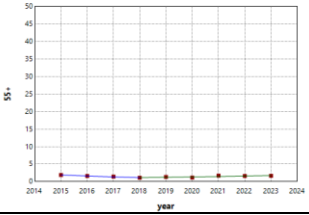 | 2015-2018<br>**-15.7**<br>(-28.0 to -6.2)<br><br>2018-2023<br>**8.8**<br>(3.4 to 20.4) |

[+]Prevalence estimates based on weighted data
Note: **Bold** indicates significant at the $p<0.05$ level.

Supplementary Figure 4. Joinpoint regression plots for the prevalence+ of oral tobacco product use*, by age and sex**, for adult participants in the US NHIS 2015-2025

| Age Groups | Males and Females | APC% (95% CI) | Males | APC% (95% CI) |
|---|---|---|---|---|
| ≥18 years | 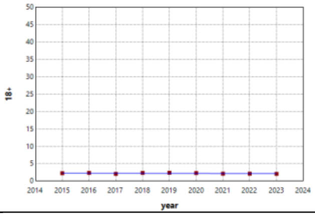 | 2015-2023<br>-0.9<br>(-3.9 to 2.3) | 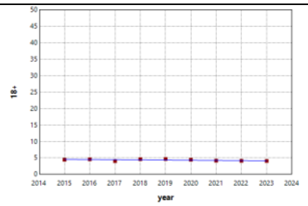 | 2015-2023<br>-1.1<br>(-3.7 to 1.6) |
| 18-24 years | 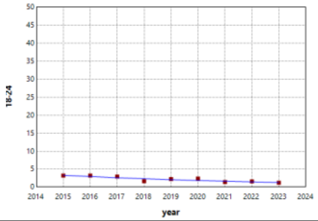 | 2015-2023<br>**-11.2**<br>(-16.1 to -5.8) | 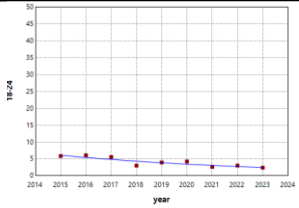 | 2015-2023<br>**-10.8**<br>(-15.5 to -5.6) |
| 25-34 years | 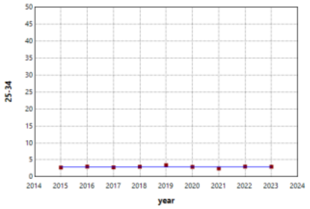 | 2015-2023<br>-0.1<br>(-2.6 to 3.0) | 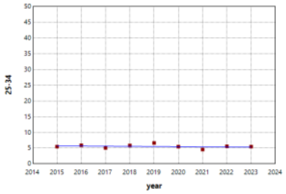 | 2015-2023<br>-0.7<br>(-3.5 to 2.2) |
| 35-54 years | 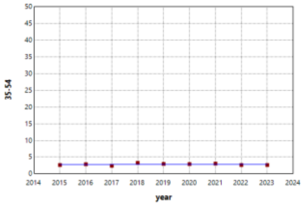 | 2015-2023<br>0.3<br>(-4.4 to 5.1) | 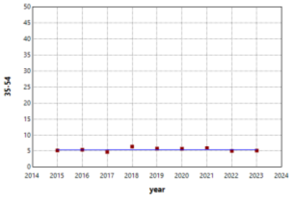 | 2015-2023<br>0.1<br>(-3.7 to 4.1) |
| ≥55 years | 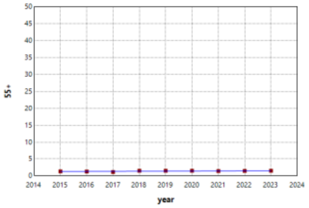 | 2015-2023<br>2.3<br>(-0.7 to 5.5) | 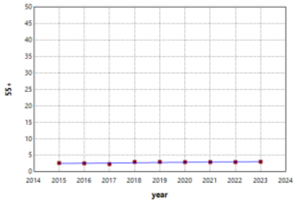 | 2015-2023<br>**2.4**<br>(0.2 to 4.7) |

[+]Prevalence estimates based on weighted data

*Oral tobacco product use includes chewing tobacco, moist snuff, and nicotine pouches

**The number of exclusive oral tobacco users among females was too low to reliably report prevalence estimates

Note: **Bold** indicates significant at the $p<0.05$ level.

Supplementary Table 1. Unweighted number (n) of people reporting cigarette use and weighted prevalence (%) estimates with 95% confidence intervals, by age and sex, for participants in the US NHIS 2015-2023

| **Year of survey** | **2015** | **2016** | **2017** | **2018** | **2019** | **2020** | **2021** | **2022** | **2023** |
|---|---|---|---|---|---|---|---|---|---|
| **Males and females** | | | | | | | | | |
| • **≥18 years** | | | | | | | | | |
| Sample size, n | 5415 | 5340 | 4015 | 3758 | 4288 | 3609 | 3405 | 3142 | 3133 |
| Prevalence, % | 15.1% | 15.5% | 14.0% | 13.7% | 14.0% | 12.5% | 11.6% | 11.6% | 10.8% |
| 95% CI | (14.6-15.7) | (14.8-16.1) | (13.3-14.6) | (13.1-14.3) | (13.4-14.5) | (11.9-13.0) | (11.1-12.1) | (10.3-11.3) | (10.3-11.3) |
| • **18-24 years** | | | | | | | | | |
| Sample size, n | 417 | 408 | 261 | 173 | 191 | 116 | 111 | 84 | 72 |
| Prevalence, % | 13.0% | 13.1% | 10.4% | 7.8% | 8.0% | 7.4% | 5.3% | 4.9% | 3.5% |
| 95% CI | (11.3-14.7) | (11.4-14.8) | (8.8-12.0) | (6.2-9.4) | (6.7-9.3) | (5.9-8.9) | (4.2-6.5) | (3.7-6.0) | (2.6-4.4) |
| • **25-34 years** | | | | | | | | | |
| Sample size, n | 1080 | 940 | 724 | 656 | 738 | 506 | 486 | 411 | 366 |
| Prevalence, % | 18.1% | 17.2% | 16.5% | 16.2% | 16.8% | 13.3% | 11.1% | 10.6% | 9.5% |
| 95% CI | (16.7-19.5) | (15.7-18.7) | (15.1-17.8) | (14.7-17.7) | (15.4-18.1) | (11.9-14.7) | (10.0-12.2) | (9.5-11.8) | (8.4-10.5) |
| • **35-54 years** | | | | | | | | | |
| Sample size, n | 2118 | 2024 | 1469 | 1403 | 1601 | 1291 | 1281 | 1145 | 1127 |
| Prevalence, % | 17.9% | 18.4% | 16.0% | 16.6% | 16.8% | 14.9% | 14.7% | 14.7% | 14.0% |
| 95% CI | (16.9-18.9) | (17.2-19.5) | (15.0-17.0) | (15.5-17.7) | (15.8-17.9) | (13.9-15.9) | (13.8-15.6) | (13.8-15.7) | 13.0-14.9) |
| • **≥55 years** | | | | | | | | | |
| Sample size, n | 1800 | 1968 | 1561 | 1526 | 1758 | 1696 | 1527 | 1502 | 1568 |
| Prevalence, % | 11.7% | 12.7% | 12.0% | 11.9% | 12.1% | 11.6% | 11.0% | 11.4% | 11.1% |
| 95% CI | (10.9-12.5) | (11.9-13.5) | (11.3-12.8) | (11.2-12.7) | (11.4-12.8) | (10.9-12.3) | (10.4-11.7) | 10.8-12.1) | (10.5-11.8) |
| **Males** | | | | | | | | | |
| • **≥18 years** | | | | | | | | | |
| Sample size, n | 2749 | 2695 | 2066 | 1943 | 2198 | 1861 | 1753 | 1648 | 1635 |
| Prevalence, % | 16.7% | 17.5% | 15.8% | 15.6% | 15.3% | 14.1% | 13.1% | 13.2% | 12.5% |
| 95% CI | (15.9-17.6) | (16.6-18.5) | (15.0-16.7) | (14.8-16.5) | (14.6-16.1) | (13.2-14.9) | (12.4-13.9) | (12.5-14.0) | (11.8-13.2) |
| • **18-24 years** | | | | | | | | | |
| Sample size, n | 229 | 213 | 148 | 98 | 98 | 69 | 64 | 56 | 46 |
| Prevalence, % | 15.0% | 14.7% | 12.0% | 8.3% | 7.8% | 9.2% | 6.2% | 6.4% | 4.4% |
| 95% CI) | (12.6-17.5) | (12.1-17.3) | (9.6-14.4) | (6.2-10.3) | (6.0-9.6) | (6.8-11.6) | (4.5-8.0) | (4.6-8.2) | (3.0-5.9) |
| • **25-34 years** | | | | | | | | | |
| Sample size, n | 564 | 471 | 395 | 340 | 396 | 291 | 268 | 242 | 203 |
| Prevalence, % | 21.3% | 20.7% | 19.9% | 18.5% | 19.1% | 16.2% | 13.6% | 13.3% | 11.5% |
| 95% CI | (19.1-23.5) | (18.3-23.0) | (17.7-22.1) | (16.3-20.6) | (17.1-21.2) | (13.9-18.4) | (11.8-15.2) | (11.4-15.2) | (9.8-13.2) |
| • **35-54 years** | | | | | | | | | |

| | | | | | | | | | |
|---|---|---|---|---|---|---|---|---|---|
| Sample size, n | 1043 | 1014 | 766 | 740 | 810 | 670 | 691 | 618 | 599 |
| Prevalence, % | 18.3% | 19.8% | 18.1% | 18.9% | 17.9% | 16.4% | 16.8% | 16.5% | 16.0% |
| 95% CI | (17.0-19.6) | (18.3-21.3) | (16.6-19.5) | (17.3-20.4) | (16.5-19.3) | (14.9-17.8) | (15.5-18.1) | (15.1-18.0) | (14.7-17.4) |
| **• ≥55 years** | | | | | | | | | |
| Sample size, n | 913 | 997 | 757 | 765 | 894 | 831 | 730 | 732 | 787 |
| Prevalence, % | 13.5% | 14.7% | 13.0% | 13.7% | 13.6% | 12.6% | 11.9% | 12.5% | 12.6% |
| 95% CI | (12.2-14.7) | (13.5-15.9) | (11.8-14.1) | (12.6-14.8) | (12.6-14.6) | (11.6-13.7) | (10.9-12.9) | (11.5-13.5) | (11.7-13.5) |
| **Females** | | | | | | | | | |
| **• ≥18 years** | | | | | | | | | |
| Sample size, n | 2666 | 2645 | 1949 | 1815 | 2090 | 1748 | 1652 | 1494 | 1498 |
| Prevalence, % | 13.6% | 13.5% | 12.2% | 12.0% | 12.7% | 11.0% | 10.1% | 10.0% | 9.2% |
| 95% CI | (12.9-14.2) | (12.8-14.3) | (11.4-13.0) | (11.2-12.7) | (12.0-13.4) | (10.3-11.6) | (9.5-10.7) | (9.4-10.7) | (8.6-9.8) |
| **• 18-24 years** | | | | | | | | | |
| Sample size, n | 188 | 195 | 113 | 75 | 93 | 47 | 47 | 28 | 26 |
| Prevalence, % | 11.0% | 11.5% | 8.8% | 7.3% | 8.2% | 5.5% | 4.5% | 3.3% | 2.5% |
| 95% CI | (8.8-13.2) | (9.4-13.7) | (6.8-10.8) | (5.1-9.5) | (6.3-10.1) | (3.7-7.4) | (3.0-5.9) | (1.9-4.7) | (1.5-3.6) |
| **• 25-34 years** | | | | | | | | | |
| Sample size, n | 516 | 469 | 329 | 316 | 342 | 215 | 219 | 169 | 163 |
| Prevalence, % | 15.0% | 13.9% | 13.0% | 14.0% | 14.4% | 10.5% | 8.6% | 8.0% | 7.5% |
| 95% CI | (13.3-16.7) | (12.2-15.5) | (11.4-14.7) | (12.1-15.9) | (12.6-16.1) | (8.9-12.0) | (7.3-9.8) | (6.6-9.6) | (6.2-8.7) |
| **• 35-54 years** | | | | | | | | | |
| Sample size, n | 1075 | 1010 | 703 | 663 | 791 | 621 | 590 | 527 | 528 |
| Prevalence, % | 17.5% | 17.0% | 14.0% | 14.4% | 15.8% | 13.5% | 12.7% | 12.9% | 11.9% |
| 95% CI | (16.2-18.8) | (15.6-18.4) | (12.7-15.3) | (13.0-15.7) | (14.5-17.1) | (12.2-14.7) | (11.6-13.8) | (11.7-14.1) | (10.7-13.0) |
| **• ≥55 years** | | | | | | | | | |
| Sample size, n | 887 | 971 | 804 | 761 | 864 | 865 | 797 | 770 | 781 |
| Prevalence, % | 10.2% | 11.0% | 11.2% | 10.4% | 10.8% | 10.7% | 10.2% | 10.5% | 9.8% |
| 95% CI | (9.3-11.1) | (10.1-11.9) | 10.3-12.2) | (9.5-11.3) | (10.0-11.7) | (9.8-11.6) | (9.4-11.0) | (9.6-11.4) | (9.0-10.6) |

Supplementary Table 2. Unweighted number (n) of people reporting other combustible tobacco use* and weighted prevalence (%) estimates with 95% confidence intervals, by age and sex, for participants in the US NHIS 2015-2023

| **Year of survey** | **2015** | **2016** | **2017** | **2018** | **2019** | **2020** | **2021** | **2022** | **2023** |
|---|---|---|---|---|---|---|---|---|---|
| **Males and females** | | | | | | | | | |
| • **≥18 years** | | | | | | | | | |
| Sample size, n | 1256 | 1455 | 1143 | 1108 | 1260 | 1119 | 1133 | 1048 | 1160 |
| Prevalence, % | 4.2% | 4.7% | 4.5% | 4.5% | 4.3% | 4.2% | 4.2% | 4.3% | 4.4% |
| 95% CI | (3.9-4.6) | (4.4-5.1) | (4.1-4.8) | (4.2-4.8) | (4.0-4.6) | (3.9-4.5) | (3.9-4.5) | (4.0-4.6) | (4.1-4.7) |
| • **18-24 years** | | | | | | | | | |
| Sample size, n | 195 | 197 | 152 | 112 | 114 | 99 | 81 | 64 | 61 |
| Prevalence, % | 6.7% | 6.0% | 6.0% | 5.3% | 5.0% | 5.7% | 4.2% | 4.0% | 3.2% |
| 95% CI | (5.5-8.0) | (4.7-7.2) | (4.9-7.1) | (3.9-6.6) | (3.9-6.2) | (4.4-7.0) | (3.1-5.2) | (3.0-5.1) | (2.3-4.2) |
| • **25-34 years** | | | | | | | | | |
| Sample size, n | 304 | 331 | 275 | 260 | 248 | 249 | 287 | 235 | 289 |
| Prevalence, % | 5.9% | 6.8% | 6.6% | 6.5% | 5.5% | 6.5% | 6.9% | 6.3% | 7.5% |
| 95% CI | (5.0-6.8) | (5.8-7.8) | (5.6-7.6) | (5.6-7.3) | (4.7-6.4) | (5.5-7.5) | (6.0-7.8) | (5.4-7.2) | (6.5-8.4) |
| • **35-54 years** | | | | | | | | | |
| Sample size, n | 409 | 488 | 359 | 351 | 459 | 405 | 396 | 366 | 385 |
| Prevalence, % | 4.1% | 4.9% | 4.3% | 4.6% | 4.9% | 4.5% | 4.5% | 4.8% | 4.9% |
| 95% CI | (3.5-4.6) | (4.3-5.5) | (3.8-4.9) | (4.0-5.2) | (4.4-5.4) | (3.9-5.0) | (4.0-5.0) | (4.2-5.3) | (4.3-5.4) |
| • **≥55 years** | | | | | | | | | |
| Sample size, n | 348 | 439 | 357 | 385 | 439 | 266 | 369 | 383 | 425 |
| Prevalence, % | 2.7% | 3.2% | 3.1% | 3.2% | 2.9% | 2.4% | 2.7% | 3.0% | 3.0% |
| 95% CI | (2.3-3.1) | (2.8-3.6) | (2.7-3.4) | (2.8-3.6) | (2.6-3.2) | (2.1-2.7) | (2.4-3.0) | (2.7-3.4) | (2.7-3.3) |
| **Males** | | | | | | | | | |
| • **≥18 years** | | | | | | | | | |
| Sample size, n | 1003 | 1177 | 944 | 910 | 1021 | 941 | 898 | 859 | 961 |
| Prevalence, % | 7.0% | 8.2% | 7.8% | 7.7% | 7.2% | 7.1% | 7.0% | 7.2% | 7.5% |
| 95% CI | (6.4-7.6) | (7.6-8.9) | (7.2-8.4) | (7.0-8.3) | (6.7-7.7) | (6.5-7.7) | (6.5-7.5) | (6.6-7.7) | (7.0-8.0) |
| • **18-24 years** | | | | | | | | | |
| Sample size, n | 125 | 152 | 119 | 85 | 74 | 74 | 55 | 49 | 48 |
| Prevalence, % | 8.6% | 9.3% | 9.5% | 7.5% | 6.6% | 8.4% | 5.8% | 6.1% | 5.2% |
| 95% CI | (6.6-10.5) | (7.3-11.4) | (7.5-11.5) | (5.2-9.7) | (4.8-8.4) | (6.2-10.6) | (4.0-7.5) | (4.2-8.0) | (3.5-6.9) |
| • **25-34 years** | | | | | | | | | |
| Sample size, n | 237 | 245 | 216 | 199 | 190 | 194 | 215 | 181 | 224 |
| Prevalence, % | 9.7% | 10.9% | 10.7% | 10.3% | 8.7% | 10.1% | 10.9% | 9.8% | 12.0% |
| 95% CI | (8.1-11.4) | (9.1-12.6) | (8.9-12.5) | (8.7-11.8) | (7.1-10.3) | (8.4-11.7) | (9.3-12.5) | (8.2-11.4) | (10.4-13.6) |
| • **35-54 years** | | | | | | | | | |

| | | | | | | | | | |
|---|---|---|---|---|---|---|---|---|---|
| Sample size, n | 326 | 399 | 295 | 289 | 364 | 341 | 311 | 297 | 306 |
| Prevalence, % | 6.8% | 8.6% | 7.6% | 7.9% | 8.2% | 7.7% | 7.5% | 8.0% | 7.9% |
| 95% CI | (5.8-7.9) | (7.5-9.7) | (6.0-8.6) | (6.7-9.0) | (7.2-9.1) | (6.7-8.6) | (6.6-8.3) | (7.0-8.9) | (6.9-8.9) |
| **• ≥55 years** | | | | | | | | | |
| Sample size, n | 315 | 381 | 314 | 337 | 393 | 332 | 317 | 332 | 383 |
| Prevalence, % | 5.3% | 6.1% | 5.9% | 6.2% | 5.7% | 4.7% | 5.1% | 5.6% | 5.8% |
| 95% CI | (4.5-6.0) | (5.3-7.0) | (5.2-6.7) | (5.4-7.0) | (5.0-6.4) | (4.1-5.4) | (4.4-5.7) | (4.9-6.3) | (5.1-6.4) |
| **Females** | | | | | | | | | |
| **• ≥18 years** | | | | | | | | | |
| Sample size, n | 253 | 278 | 199 | 198 | 239 | 178 | 235 | 189 | 199 |
| Prevalence, % | 1.6% | 1.5% | 1.4% | 1.5% | 1.5% | 1.5% | 1.6% | 1.5% | 1.4% |
| 95% CI | (1.3-1.9) | (1.2-1.7) | (1.1-1.6) | (1.3-1.8) | (1.3-1.8) | (1.2-1.7) | (1.3-1.8) | (1.1-1.8) | (1.1-1.6) |
| **• 18-24 years** | | | | | | | | | |
| Sample size, n | 70 | 45 | 33 | 27 | 40 | 25 | 26 | 15 | 13 |
| Prevalence, % | 4.8% | 2.6% | 2.4% | 3.0% | 3.4% | 3.0% | 2.5% | 1.9% | 1.2% |
| 95% CI | (3.3-6.2) | (1.5-3.7) | (1.4-3.4) | (1.5-4.4) | (2.1-4.7) | (1.7-4.3) | (1.5-3.6) | (0.8-3.0) | (0.4-2.0) |
| **• 25-34 years** | | | | | | | | | |
| Sample size, n | 67 | 86 | 59 | 61 | 58 | 55 | 72 | 54 | 65 |
| Prevalence, % | 2.3% | 2.8% | 2.5% | 2.7% | 2.3% | 2.9% | 2.9% | 2.8% | 2.8% |
| 95% CI | (1.5-3.0) | (1.9-3.7) | (1.7-3.4) | (1.9-3.6) | (1.7-3.0) | (2.0-3.8) | 2.1-3.7) | (1.8-3.8) | (2.0-3.6) |
| **• 35-54 years** | | | | | | | | | |
| Sample size, n | 83 | 89 | 64 | 62 | 95 | 64 | 85 | 69 | 79 |
| Prevalence, % | 1.4% | 1.3% | 1.3% | 1.5% | 1.7% | 1.4% | 1.7% | 1.6% | 1.8% |
| 95% CI | (1.0-1.8) | (0.9-1.6) | (0.9-1.6) | (1.0-1.9) | (1.3-2.1) | (1.0-1.8) | (1.2-2.1) | (1.2-2.1) | (1.3-2.2) |
| **• ≥55 years** | | | | | | | | | |
| Sample size, n | 33 | 58 | 43 | 48 | 46 | 34 | 52 | 51 | 42 |
| Prevalence, % | 0.5% | 0.7% | 0.6% | 0.7% | 0.5% | 0.4% | 0.7% | 0.7% | 0.5% |
| 95% CI | (0.3-0.7) | (0.4-0.9) | (0.4-0.8) | (0.4-0.9) | (0.3-0.7) | (0.3-0.6) | (0.5-0.9) | (0.5-0.9) | (0.3-0.7) |

*Other combustible tobacco use includes cigars, pipe tobacco, and hookah tobacco

Supplementary Table 3. Unweighted number (n) of people reporting electronic cigarette use and weighted prevalence (%) estimates with 95% confidence intervals, by age and sex, for participants in the US NHIS 2015-2023

| **Year of survey** | **2015** | **2016** | **2017** | **2018** | **2019** | **2020** | **2021** | **2022** | **2023** |
|---|---|---|---|---|---|---|---|---|---|
| **Males and females** | | | | | | | | | |
| • **≥18 years** | | | | | | | | | |
| Sample size, n | 1029 | 983 | 740 | 783 | 1201 | 869 | 1110 | 1272 | 1513 |
| Prevalence, % | 3.5% | 3.2% | 2.8% | 3.2% | 4.5% | 3.7% | 4.5% | 6.0% | 6.5% |
| 95% CI | (3.2-3.7) | (2.9-3.5) | (2.5-3.1) | (3.0-3.5) | (4.1-4.8) | (3.4-4.0) | (4.2-4.9) | (5.6-6.4) | (6.1-6.9) |
| • **18-24 years** | | | | | | | | | |
| Sample size, n | 140 | 133 | 128 | 157 | 204 | 160 | 216 | 250 | 249 |
| Prevalence, % | 5.2% | 4.7% | 5.2% | 7.6% | 9.3% | 9.4% | 11.0% | 15.3% | 13.4% |
| 95% CI | (4.2-6.2) | (3.5-5.9) | (3.9-6.5) | (6.1-9.1) | (7.8-10.7) | (7.7-11.1) | (9.3-12.6) | (13.3-17.3) | (11.6-15.2) |
| • **25-34 years** | | | | | | | | | |
| Sample size, n | 232 | 205 | 196 | 207 | 344 | 250 | 331 | 387 | 494 |
| Prevalence, % | 4.3% | 4.2% | 4.7% | 5.4% | 7.4% | 6.1% | 7.8% | 10.6% | 12.6% |
| 95% CI | (3.5-5.0) | (3.4-5.0) | (3.8-5.5) | (4.6-6.2) | (6.5-8.4) | (5.3-7.0) | (6.8-8.7) | (9.5-11.8) | (11.4-13.8) |
| • **35-54 years** | | | | | | | | | |
| Sample size, n | 401 | 396 | 242 | 255 | 436 | 311 | 364 | 434 | 533 |
| Prevalence, % | 4.0% | 3.8% | 2.5% | 2.8% | 4.6% | 3.5% | 4.0% | 5.7% | 6.5% |
| 95% CI | (3.5-4.5) | (3.3-4.3) | (2.1-3.0) | (2.4-3.2) | (4.1-5.1) | (3.0-4.0) | (3.5-4.5) | (5.0-6.3) | (5.9-7.2) |
| • **≥55 years** | | | | | | | | | |
| Sample size, n | 256 | 249 | 174 | 164 | 217 | 148 | 199 | 201 | 237 |
| Prevalence, % | 1.9% | 1.6% | 1.4% | 1.2% | 1.4% | 1.0% | 1.6% | 1.5% | 1.7% |
| 95% CI | (1.6-2.2) | (1.3-1.8) | (1.1-1.6) | (1.0-1.4) | (1.2-1.6) | (0.8-1.2) | (1.4-1.9) | (1.3-1.7) | (1.5-2.0) |
| **Males** | | | | | | | | | |
| • **≥18 years** | | | | | | | | | |
| Sample size, n | 557 | 525 | 397 | 471 | 686 | 496 | 577 | 666 | 809 |
| Prevalence, % | 4.3% | 3.8% | 3.3% | 4.3% | 5.5% | 4.6% | 5.1% | 6.9% | 7.6% |
| 95% CI | (3.9-4.8) | (3.4-4.3) | (2.8-3.7) | (3.8-4.8) | (5.0-6.0) | (4.2-5.1) | (4.7-5.6) | (6.3-7.5) | (7.0-8.3) |
| • **18-24 years** | | | | | | | | | |
| Sample size, n | 95 | 93 | 80 | 107 | 120 | 100 | 113 | 138 | 139 |
| Prevalence, % | 7.8% | 7.1% | 6.4% | 10.2% | 10.8% | 12.2% | 11.6% | 16.8% | 14.7% |
| 95% CI | (5.9-9.7) | (4.9-9.3) | (4.4-8.4) | (7.8-12.6) | (8.6-13.1) | (9.6-14.9) | (9.1-14.1) | (14.0-19.7) | (12.3-17.1) |
| • **25-34 years** | | | | | | | | | |
| Sample size, n | 152 | 123 | 121 | 135 | 224 | 157 | 198 | 224 | 292 |
| Prevalence, % | 6.1% | 5.4% | 5.8% | 7.7% | 9.4% | 7.9% | 10.0% | 12.9% | 15.6% |
| 95% CI | (4.7-7.5) | (4.1-6.7) | (4.6-7.0) | (6.2-9.1) | (8.0-10.9) | (6.6-9.3) | (8.5-11.5) | (11.0-14.7) | (13.7-17.4) |
| • **35-54 years** | | | | | | | | | |

| | | | | | | | | | |
|---|---|---|---|---|---|---|---|---|---|
| Sample size, n | 202 | 206 | 123 | 150 | 244 | 180 | 178 | 227 | 272 |
| Prevalence, % | 4.5% | 4.2% | 2.8% | 3.4% | 5.6% | 4.2% | 4.2% | 6.3% | 7.3% |
| 95% CI | (3.7-5.3) | (3.4-5.0) | (2.2-3.5) | (2.8-4.1) | (4.8-6.4) | (3.5-5.0) | (3.5-4.8) | (5.4-7.2) | (6.3-8.4) |
| • **≥55 years** | | | | | | | | | |
| Sample size, n | 108 | 103 | 73 | 79 | 98 | 59 | 88 | 77 | 106 |
| Prevalence, % | 1.9% | 1.5% | 1.3% | 1.3% | 1.5% | 0.9% | 1.5% | 1.4% | 1.8% |
| 95% CI | (1.4-2.4) | (1.1-1.9) | (1.0-1.6) | (1.0-1.7) | (1.1-1.8) | (0.6-1.2) | (1.1-1.9) | (1.1-1.7) | (1.4-2.2) |
| **Females** | | | | | | | | | |
| • **≥18 years** | | | | | | | | | |
| Sample size, n | 472 | 458 | 343 | 312 | 515 | 373 | 533 | 606 | 704 |
| Prevalence, % | 2.6% | 2.6% | 2.4% | 2.3% | 3.5% | 2.8% | 4.0% | 5.2% | 5.5% |
| 95% CI | (2.3-3.0) | (2.2-2.9) | (2.0-2.7) | (2.0-2.6) | (3.1-3.9) | (2.4-3.2) | (3.6-4.4) | (4.7-5.7) | (5.0-5.9) |
| • **18-24 years** | | | | | | | | | |
| Sample size, n | 45 | 40 | 48 | 50 | 84 | 60 | 103 | 112 | 110 |
| Prevalence, % | 2.5% | 2.3% | 4.0% | 4.9% | 7.7% | 6.5% | 10.3% | 13.7% | 12.1% |
| 95% CI | (1.7-3.3) | (1.4-3.1) | (2.3-5.8) | (3.3-6.6) | (5.8-9.5) | (4.5-8.6) | (8.0-12.6) | (10.9-16.6) | (9.6-14.6) |
| • **25-34 years** | | | | | | | | | |
| Sample size, n | 80 | 82 | 75 | 72 | 120 | 93 | 133 | 163 | 202 |
| Prevalence, % | 2.5% | 3.1% | 3.6% | 3.2% | 5.4% | 4.3% | 5.6% | 8.4% | 9.5% |
| 95% CI | (1.7-3.3) | (2.1-4.0) | (2.6-4.6) | (2.3-4.0) | (4.3-6.6) | (3.3-5.3) | (4.5-6.6) | (7.0-9.9) | (8.1-11.0) |
| • **35-54 years** | | | | | | | | | |
| Sample size, n | 199 | 190 | 119 | 105 | 192 | 131 | 186 | 207 | 261 |
| Prevalence, % | 3.6% | 3.5% | 2.2% | 2.3% | 3.7% | 2.8% | 3.8% | 5.0% | 5.7% |
| 95% CI | (3.0-4.2) | (2.8-4.2) | (1.8-2.7) | (1.8-2.8) | (3.1-4.3) | (2.2-3.4) | (3.2-4.5) | (4.2-5.8) | (4.9-6.5) |
| • **≥55 years** | | | | | | | | | |
| Sample size, n | 148 | 146 | 101 | 85 | 119 | 89 | 111 | 124 | 131 |
| Prevalence, % | 1.9% | 1.6% | 1.4% | 1.1% | 1.3% | 1.2% | 1.7% | 1.6% | 1.7% |
| 95% CI | (1.7-2.3) | (1.3-2.0) | (1.1-1.8) | (0.8-1.4) | (1.1-1.6) | (0.8-1.5) | (1.3-2.1) | (1.3-1.9) | (1.4-2.0) |

Supplementary Table 4. Unweighted number (n) of people reporting oral tobacco use* and weighted prevalence (%) estimates with 95% confidence intervals, by age and sex**, for participants in the US NHIS 2015-2023

| **Year of survey** | **2015** | **2016** | **2017** | **2018** | **2019** | **2020** | **2021** | **2022** | **2023** |
|---|---|---|---|---|---|---|---|---|---|
| **Males and females** | | | | | | | | | |
| • **≥18 years** | | | | | | | | | |
| Sample size, n | 675 | 849 | 591 | 619 | 697 | 606 | 568 | 528 | 578 |
| Prevalence, % | 2.3% | 2.4% | 2.1% | 2.4% | 2.4% | 2.3% | 2.1% | 2.1% | 2.1% |
| 95% CI | (1.9-2.5) | (2.1-2.6) | (1.8-2.3) | (2.1-2.6) | (2.2-2.7) | (2.1-2.6) | (1.9-2.4) | (1.9-2.4) | (1.9-2.3) |
| • **18-24 years** | | | | | | | | | |
| Sample size, n | 87 | 112 | 76 | 45 | 42 | 42 | 28 | 25 | 26 |
| Prevalence, % | 3.2% | 3.2% | 2.9% | 1.6% | 2.2% | 2.4% | 1.4% | 1.6% | 1.2% |
| 95% CI | (2.2-4.1) | (2.4-4.0) | (2.1-3.7) | (1.1-2.1) | (1.4-3.0) | (1.5-3.2) | (0.8-2.0) | (0.9-2.2) | (0.7-1.7) |
| • **25-34 years** | | | | | | | | | |
| Sample size, n | 154 | 174 | 130 | 136 | 153 | 131 | 107 | 110 | 125 |
| Prevalence, % | 2.7% | 3.0% | 2.8% | 3.0% | 3.4% | 2.9% | 2.4% | 3.0% | 3.0% |
| 95% CI | (2.1-3.3) | (2.4-3.6) | (2.2-3.3) | (2.4-3.6) | (2.8-4.1) | (2.3-3.6) | (1.9-2.9) | (2.4-3.6) | (2.3-3.6) |
| • **35-54 years** | | | | | | | | | |
| Sample size, n | 285 | 336 | 235 | 257 | 298 | 236 | 240 | 204 | 206 |
| Prevalence, % | 2.6% | 2.9% | 2.4% | 3.3% | 3.0% | 2.9% | 3.1% | 2.6% | 2.6% |
| 95% CI | (2.2-3.1) | (2.5-3.3) | (2.0-2.8) | (2.8-3.8) | (2.6-3.4) | (2.5-3.4) | (2.6-3.5) | (2.2-3.1) | (2.2-3.1) |
| • **≥55 years** | | | | | | | | | |
| Sample size, n | 149 | 227 | 150 | 181 | 204 | 197 | 193 | 189 | 221 |
| Prevalence, % | 1.3% | 1.3% | 1.1% | 1.5% | 1.5% | 1.5% | 1.4% | 1.5% | 1.5% |
| 95% CI | (1.0-1.7) | (1.1-1.5) | (0.9-1.4) | (1.2-1.8) | (1.2-1.8) | (1.2-1.8) | (1.2-1.7) | (1.2-1.7) | (1.3-1.8) |
| **Males** | | | | | | | | | |
| • **≥18 years** | | | | | | | | | |
| Sample size, n | 631 | 797 | 554 | 583 | 654 | 567 | 528 | 494 | 541 |
| Prevalence, % | 4.4% | 4.6% | 4.0% | 4.7% | 4.7% | 4.5% | 4.2% | 4.1% | 4.1% |
| 95% CI | (3.9-5.0) | (4.1-5.0) | (3.6-4.5) | (4.2-5.2) | (4.2-5.2) | (4.0-5.0) | (3.7-4.6) | (3.6-4.6) | (3.6-4.5) |
| • **18-24 years** | | | | | | | | | |
| Sample size, n | 79 | 106 | 72 | 42 | 38 | 37 | 27 | 24 | 26 |
| Prevalence, % | 5.8% | 6.1% | 5.56% | 3.0% | 3.9% | 4.2% | 2.7% | 3.0% | 2.4% |
| 95% CI | (4.0-7.7) | (4.6-7.6) | (4.0-7.2) | (2.0-4.0) | (2.4-5.5) | (2.6-5.8) | (1.4-3.9) | (1.7-4.3) | (1.3-3.4) |
| • **25-34 years** | | | | | | | | | |
| Sample size, n | 151 | 168 | 120 | 131 | 144 | 124 | 96 | 102 | 116 |
| Prevalence, % | 5.5% | 5.9% | 5.1% | 5.9% | 6.6% | 5.5% | 4.5% | 5.6% | 5.5% |
| 95% CI | (4.2-6.7) | (4.7-7.1) | (4.0-6.1) | (4.7-7.0) | (5.3-8.0) | (4.2-6.7) | (3.6-5.5) | (4.4-6.8) | (4.3-6.6) |
| • **35-54 years** | | | | | | | | | |

| | | | | | | | | | |
|---|---|---|---|---|---|---|---|---|---|
| Sample size, n<br>Prevalence, %<br>95% CI | 271<br>5.2%<br>(4.3-6.1) | 316<br>5.4%<br>(4.6-6.2) | 225<br>4.7%<br>(4.0-5.4) | 245<br>6.4%<br>(5.4-7.3) | 287<br>5.8%<br>(5.0-6.6) | 226<br>5.8%<br>(4.9-6.7) | 226<br>5.9%<br>(5.0-6.8) | 192<br>5.0%<br>(4.2-5.9) | 197<br>5.1%<br>(4.3-6.0) |
| **• ≥55 years** | | | | | | | | | |
| Sample size, n<br>Prevalence, %<br>95% CI | 130<br>2.6%<br>(1.9-3.3) | 207<br>2.5%<br>(2.1-3.0) | 137<br>2.3%<br>(1.8-2.8) | 165<br>3.0%<br>(2.4-3.5) | 185<br>3.0%<br>(2.4-3.5) | 180<br>2.9%<br>(2.4-3.5) | 179<br>2.9%<br>(2.4-3.4) | 176<br>2.9%<br>(2.4-3.4) | 202<br>3.0%<br>(2.5-3.5) |

*Oral tobacco includes chewing tobacco, moist snuff, and nicotine pouches

**The number of exclusive oral tobacco users among females was too low to reliably report prevalence estimates

© 2026 Mok Y. et al.
